# The relationship between patient and practitioner expectations and preferences and clinical outcomes in a trial of exercise and acupuncture for knee osteoarthritis

**DOI:** 10.1016/j.ejpain.2009.06.010

**Published:** 2010-04

**Authors:** Nadine E. Foster, Elaine Thomas, Jonathan C. Hill, Elaine M. Hay

**Affiliations:** Arthritis Research Campaign National Primary Care Centre, Primary Care Sciences, Keele University, Keele, Staffordshire ST5 5BG, United Kingdom

**Keywords:** Preference, Expectation, Physiotherapy, Acupuncture, Exercise

## Abstract

We investigated the relationship between patient and therapist preferences and expectations and clinical outcomes in a trial of exercise and acupuncture for clinical knee osteoarthritis.

352 Patients were randomised to advice and exercise or advice and exercise plus true or non-penetrating acupuncture. Before randomisation, patients recorded their general outcome expectations, treatment-specific preferences and expectations. Clinical outcome was (a) change scores on the Western Ontario and McMaster Osteoarthritis Index (WOMAC) and (b) treatment response according to the OMERACT-OARSI criteria. Physiotherapists recorded their treatment expectations and preferences for each patient following an assessment prior to randomisation. We investigated the relationship between (a) patient, (b) therapist and (c) matched patient–therapist preferences and expectations on clinical outcomes using univariate and multivariate analyses.

There was no significant relationship between patients’ treatment preferences and clinical outcomes at 6 or 12 months nor between patients’ expectations and pain (WOMAC) at 6 or 12 months. Using our secondary outcome (OMERART-OARSI), those who received the treatment for which they had high expectations of benefit were almost twice as likely to be classified as a treatment responder at 6 months (odds ratio (OR) 1.7 (95% Confidence Interval 1.06, 2.79)) and 12 months (OR) 1.9 (1.13, 3.13). Therapists’ preferences and expectations for individual patients did not add further explanation of outcomes.

There was no evidence of a relationship between patients’ treatment preferences or expectations and pain reduction. We found weak evidence, from secondary outcomes, that patients’ expectations, both general and treatment-specific, are related to clinical outcome from exercise and acupuncture.

## 1 Introduction

In addition to specific effects of treatments, many non-specific, or ‘placebo’, effects have been suggested. These include factors such as patients’ attitudes and beliefs, treatment preferences and expectations, the nature and setting of the intervention, as well as practitioner’s characteristics including their therapeutic style, attitudes and beliefs, and treatment preferences (Crow et al., 1999, Ernst, 2001, Feinstein, 2002, Foster, 2007). These ‘meaning and context effects’ are emphasised within the complementary medicine literature (Bausell et al., 2005, Manheimer et al., 2007, Kaptchuk et al., 2008). Patients who express preferences for specific treatments may have different clinical characteristics from those who do not, influencing prognosis and outcome.

A review of different health conditions found little evidence for a ‘preference effect’ (King et al., 2005), yet there is some evidence for a relationship between treatment preferences and expectations and clinical outcomes in musculoskeletal pain (Van der Windt et al., 2000, Klaber-Moffett et al., 2005, Heymans et al., 2006, Johnson et al., 2007, Preference Collaborative Review Group, 2008), and complementary therapies (Kalauokalani et al., 2001, Linde et al., 2007). Some argue that it is the ability of the patient to articulate a treatment preference, not the preference itself, that is associated with better outcomes (Thomas et al., 2004). Others conclude no relationship between preferences or expectations and outcomes (Moffett et al., 1999, Ersek et al., 2003).

Recently attention has shifted to healthcare practitioners (Di Blasi et al., 2001, King et al., 2005, Pincus et al., 2006), since their preferences and expectations might influence patient assessment, the importance placed on assessment findings, enthusiasm and confidence in treatments and patient communication (Foster, 2007). Finniss and Benedetti (2005) raise the possibility that clinician’s words can have powerful effects on patient’s expectations, subsequent neurobiological changes and pain levels but empirical data are lacking. Practitioners hold a wide range of beliefs about pain that correlate with their recommendations to patients (Coudeyre et al., 2006, Poiraudeau et al., 2006, Pincus et al., 2007, Bishop et al., 2008). There is evidence that patients’ and practitioners’ expectations conflict (Montgomery and Fahey, 2001, Parsons et al., 2007) and that practitioners’ preferences affect the external and internal validity of clinical trials (King et al., 2005). Few studies have investigated the relationship between practitioners’ expectations or preferences and patient outcomes (Galer et al., 1997, Turner et al., 2002, King et al., 2005, Stewart et al., 2008).

A randomised trial tested the additional benefit of acupuncture to advice and exercise for knee osteoarthritis (Foster et al., 2007). There were no significant differences in pain reduction between advice and exercise, advice and exercise plus true acupuncture or non-penetrating acupuncture. Small additional benefits in pain intensity and unpleasantness were observed in both acupuncture groups, making it unlikely that acupuncture effects are explained by specific physiological mechanisms of needling. In this paper, we address whether there are relationships between:

(1) patients’ preferences and expectations and clinical outcomes,
(2) physiotherapists’ preferences and expectations and patients’ clinical outcomes,
(3) ‘matched’ patients’ and therapists’ preferences and expectations and clinical outcomes.

## 2 Methods

### 2.1 Design and setting

Full details of the trial, interventions and clinical outcomes are available (Foster et al., 2007). The trial was conducted in 37 physiotherapy centres in the UK between November 2003 and October 2005. The study was approved by the UK West Midlands multicentre research ethics committee and by 13 local ethics committees. This paper reports the results from the analyses of the preferences and expectations data within the trial, gathered to address *a priori* research questions specified in the trial protocol (Hay et al., 2004). Data were collected at baseline, 6 months and 12 months.

### 2.2 Participants: recruitment and data collection

Full details of recruitment method, inclusion and exclusion criteria have been reported previously (Hay et al., 2004, Foster et al., 2007). Potentially eligible participants were posted information about the study and a standard proforma used to collect information from a subsequent telephone screen. All potentially eligible patients were asked three questions regarding treatment preference and expectation. Responses to the question “If you had a free choice what treatment would you choose?” were recorded verbatim. Strength of the expectation that (i) advice and exercise and (ii) acupuncture would improve their knee problem were recorded using an 11 point numerical rating scale (NRS) were 0 represented lowest expectation and 10 represented the highest expectation.

For those patients eligible and consenting to take part in the trial (*n* = 352: 116 to advice and exercise, 117 to advice and exercise plus true acupuncture, and 119 to advice and exercise plus non-penetrating acupuncture), detailed information on their treatment preferences and strength of expectations were recorded prior to randomisation. They were asked to state if they had a treatment preference, and if so, which treatment they would choose, and to indicate the strength of their treatment preference for advice and exercise and acupuncture (“strongly prefer” to “strongly not prefer”). General outcome expectation was recorded (11 point NRS) and treatment-specific expectation for advice and exercise and acupuncture (overall strength (11 point NRS) and specifically for pain (“great help”, “some help”, “little help” and “no help”). All questions about preferences and expectations were independent of randomisation and had no influence on it. The questions are given in Appendix 1.

The primary clinical outcome measure was 6 months change in the pain subscale score of the Western Ontario and McMaster Universities osteoarthritis index (WOMAC Likert 3.0) (Bellamy, 1996). The secondary outcome was a clinically significant response at 6 months according to criteria from the outcome measures in Rheumatology and Osteoarthritis Research Society international initiative (OMERACT-OARSI) (Pham et al., 2003, Pham et al., 2004). The OMERACT-OARSI criteria uses change scores in pain and function (here using WOMAC scores converted to 0–100 points) and a global change measure (five options: “much better” to “much worse”) to define those achieving “high improvement” (change in score of ⩾50% and an absolute change of ⩾20 points on either pain or function) or “improvement” (2 from 3 of change in score of ⩾20% and an absolute change of ⩾10 on pain or function or global change of “much better” or “better”) which are combined to define the “responder” group. Follow-up at the main outcome time point of 6 months was 94% and the proportion of treatment responders at this point was similar in the three intervention groups (43%, 50%, 52%, respectively).

### 2.3 Interventions

Participants were told they would receive physiotherapy advice and exercise and “may receive acupuncture, using one of two different types of acupuncture needle” without specifying the needles’ mode of action (penetrating compared with non-penetrating) to maximise the effectiveness of blinding. In the advice and exercise group, participants received an advice leaflet, and an individualised exercise programme, oriented towards lower limb strengthening, stretching, and balance, which was progressed and supervised in up to six treatment sessions. In the group receiving advice and exercise plus true acupuncture, participants also received acupuncture on traditional Chinese acupuncture points, using between 6 and 10 acupuncture points from 16 commonly used local and distal points. In the group receiving advice and exercise plus non-penetrating acupuncture (Streitberger and Kleinhenz, 1998), participants also received acupuncture through needles with a blunt tip. The same protocol was used as for true acupuncture, thus participants had the same contact time and interaction between therapist and patient, the same manual contact during the search for acupuncture points and the same attention to elicited sensations. Patients reported all treatments to be highly credible with no differences between groups (Foster et al., 2007). Researchers who collected, entered, and analysed data were blinded to treatment allocation.

### 2.4 Physiotherapists

Interventions were delivered by 67 physiotherapists in 37 NHS physiotherapy centres trained in acupuncture to at least minimum national standards for membership of the Acupuncture Association of Chartered Physiotherapists. Two thirds of the physiotherapists had been qualified for more than 10 years and over half had been using acupuncture for more than 3 years. By necessity the physiotherapists delivering the interventions were not blind to allocation. All patients had an initial clinical physiotherapy assessment of up to 40 min duration, during which the physiotherapist identified and recorded the acupuncture points to be used should the patient be randomised to receive acupuncture. Following this assessment, each physiotherapist telephoned the Research Centre to randomise the patient in the trial. During this telephone call, the physiotherapist answered questions about their own treatment expectations and preferences for that individual patient. They were asked to report their general outcome expectation for the patient (NRS), to state if they had a treatment preference for the individual patient, and if so, which treatment they would choose and to record how much they expected each treatment within the trial to help the individual patient’s knee pain (“great help”, “some help”, “little help” and “no help”). The questions are reproduced in Appendix 2.

### 2.5 Statistical analysis

#### 2.5.1 Patients’ preferences and expectations

Baseline characteristics were compared for those participants who did and did not report a treatment preference. Participants’ baseline treatment preference, strength of preference data and changes in preference and strength of preference from baseline to 6 months were described according to randomised treatment group. Patient expectations of benefit (general outcome expectation, expectation of the benefit from each treatment for their pain, and strength of expectation) were examined at baseline overall and for each treatment group. Data on the strength of overall and treatment-specific expectations, both measured by an 11 point NRS, were dichotomised at the median, as used previously (Kalauokalani et al., 2001) to define groups with “higher” or “lower” expectation.

Regression models assessed the ability of baseline treatment preference (no or yes), strength of treatment preferences and expectations (“higher” or “lower”) to predict change in WOMAC pain (linear regression) and OMERACT-OARSI responder (logistic regression) at 6 and 12 months. Each model was then adjusted for baseline WOMAC pain, age, gender, duration of problem and randomised treatment group.

In addition to examining the general predictive ability of these measures of preference and expectation, we formed two additional predictor variables for use in statistical modelling, as follows: (i) *Treatment choice*: We assembled a group of participants whose preferred treatment matched the actual treatment group they were randomly allocated to (we classified the choice of acupuncture in those allocated to receive non-penetrating acupuncture as a match). The comparison group consisted of ‘unmatched’ participants i.e. those who either did not state a preferred treatment or those who stated a preferred treatment other than the one they were randomised to. (ii) *Expectation for treatment benefit*: We assembled a group of participants who reported ‘higher’ expectations for the treatment to which they were randomised. For those randomised to non-penetrating acupuncture, their expectation score for acupuncture was used. The comparison group was those who had ‘lower’ expectations for the treatment to which they were randomised.

#### 2.5.2 Therapists’ preferences and expectations

Physiotherapists’ baseline treatment preference and strength of preference data for individual participants were examined by patients’ randomised treatment group. The processes described above for matching preferences and expectations were repeated for the physiotherapist data.

#### 2.5.3 Matching of patients and therapists preferences and expectations

We assembled groups combining both patient and physiotherapist expectations/preferences with the patients’ randomised treatment allocation. For preferences; patients in whom both the patient and the physiotherapist gave a preference for their randomised treatment were compared to the remaining patients. For expectations; patients in whom both the patient and the physiotherapist reported a higher expectation of benefit from their randomised treatment were compared to other patients. Regression models, as described above, were fitted to determine whether matched treatment preferences or expectations in both patients and physiotherapists were predictive of clinical outcome at 6 and 12 months.

All statistical analyses were performed using Stata 7.0.

## 3 Results

### 3.1 Patients’ preferences and expectations

**Fig. 1:**
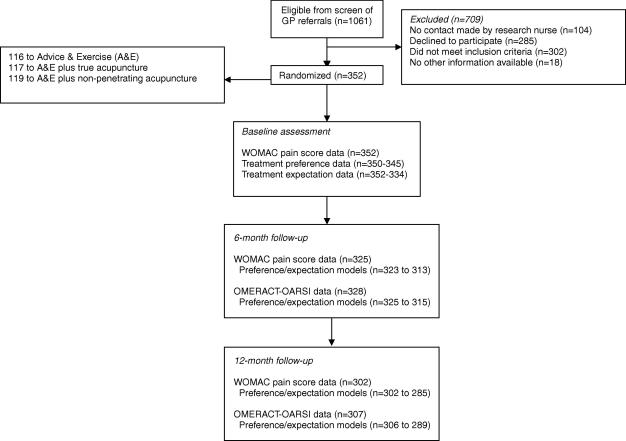
Flow chart summarising follow-up on clinical outcomes and treatment preferences and expectations.

1061 Potentially eligible participants were identified from referral forms, of whom 709 (66.8%) were ineligible or did not want to participate. Fig. 1 summarises the flow of patients and follow-up in this study.

Of the 709 who did not participate in the trial, 94 responded to the additional questions about treatment preferences and expectations; 71% reported a treatment preference of whom 40% stated exercise and 16% stated acupuncture. Other examples included hydrotherapy and heat. Using the numerical rating scale (where 0 is expectation of no benefit and 10 is expectation of being completely better), those not participating in the trial had a mean level of expectation for advice and exercise of 6.2 (2.7) and for acupuncture, 3.6 (3.4).

Of the 352 participants randomised to the trial, only 70 (20%) reported a treatment preference. Table 1 shows baseline characteristics (socio-demographic, knee specific and randomised treatment) for those with and without a treatment preference; two participants did not complete this question. Patients reporting a preference were similar to those reporting no preference, although patients who had knee symptoms for less than 1 year were more likely to have no treatment preference. Patients with and without treatment preferences were randomised equally to all treatment arms of the trial.

**Table 1:** Baseline characteristics of trial participants: comparing those with and without a treatment preference.

Baseline characteristics	With a treatment preference (*n* = 70)	Without a treatment preference (*n* = 280)
*Socio-demographic*		
Mean (SD) age (years)	65.3 (9.3)	62.7 (8.6)
Women	42 (60%)	173 (62%)
Body mass index		
Underweight or normal	16 (23%)	56 (20%)
Overweight	33 (47%)	124 (45%)
Obese	21 (30%)	97 (35%)
*Socio-economic classification*^a^		
Higher managerial or professional	8 (12%)	21 (8%)
Lower managerial or professional	11 (16%)	45 (17%)
Intermediate occupations	12 (18%)	45 (17%)
Self-employed	4 (6%)	19 (8%)
Lower supervisory or technical	4 (6%)	16 (6%)
Semi-routine	16 (24%)	65 (25%)
Routine	12 (18%)	50 (19%)
Currently employed	26 (37%)	111 (40%)

*Knee pain and function*		
Mean (SD) WOMAC pain score	9.1 (4.0)	9.1 (3.6)
Mean (SD) WOMAC function score	31.1 (13.5)	30.2 (13.0)
Total duration of knee pain (years)		
<1	23 (33%)	125 (45%)
1 to <5	25 (36%)	87 (31%)
5 to <10	8 (11%)	32 (11%)
⩾10	14 (20%)	36 (13%)
Onset of current problems		
Sudden	29 (41%)	131 (47%)
Gradual	41 (59%)	149 (53%)
Mean (SD) pain severity in past 7 days	6.3 (2.2)	5.8 (2.2)
Mean (SD) pain unpleasantness in past 7 days	6.4 (2.3)	5.8 (2.3)

*Randomised treatment*		
Advice and exercise (A&E)	23 (33%)	93 (33%)
A&E + true acupuncture	22 (31%)	93 (33%)
A&E + non-penetrating acupuncture	25 (36%)	94 (34%)

a Office of National Statistics. Standard occupational classification, 2000, vol. 2. The coding index. The Stationary Office: London, 2000:4. Office for National Statistics. The National Statistics Socio-economic classification user manual. Version 1. ONS: London, 2002:4.

At baseline, all participants were asked which treatment they would choose if they had a free choice: 10% stated advice and exercise, 13% acupuncture and 44% both. Very few other treatments were reported, with similar responses across the randomised groups (Table S1). Treatment preferences and overall strength of preferences for each treatment were similar between groups. Patients’ outcome and treatment expectations are summarised in Table S1. Patients had, on average, high expectations of improvement at baseline. Overall mean baseline levels of expectation of benefit from treatments were 5.9 (2.3) and 6.3 (2.2) for advice and exercise, and for acupuncture, respectively, and there was little difference between the three trial arms. Very few patients reported expecting the available treatments to be of ‘little’ or ‘no’ help.

Table 2 presents the unadjusted and adjusted coefficients testing the relationships between patients’ baseline treatment preferences and expectations and their clinical outcome (WOMAC pain subscale) at 6 and 12 months. There was no relationship between patients’ baseline treatment preferences and the change in their knee pain over time.

**Table 2:** Association of patients baseline treatment preferences and expectations with change in WOMAC pain score.

	Change in WOMAC pain at 6 months		Change in WOMAC pain at 12 months	
	Unadjusted mean difference (95% CI)	Adjusted^a^ mean difference (95% CI)	Unadjusted mean difference (95% CI)	Adjusted^a^ mean difference (95% CI)
*Preference for treatment received*				
Yes cf. no^b^	0.19 (−0.86, 1.25)	0.43 (−0.58, 1.43)	0.23 (−0.95, 1.41)	0.45 (−0.66, 1.56)

*Preference for A&E*				
Strongly prefer/prefer cf. no preference^b^	0.16 (−0.72, 1.04)	0.45 (−0.40, 1.31)	−0.25 (−1.24, 0.73)	−0.006 (−0.95, 0.94)
Strongly not prefer/not prefer cf. no preference^b^	−0.17 (−1.95, 1.61)	−0.97 (−2.66, 0.73)	0.21 (−1.91, 2.32)	−0.65 (−2.66, 1.35)

*Preference for acupuncture*				
Strongly prefer/prefer cf. no preference^b^	0.44 (−0.43, 1.31)	0.16 (−0.69, 1.02)	0.19 (−0.79, 1.17)	−0.19 (−1.14, 0.76)
Strongly not prefer/not prefer cf. no preference^b^	−0.91 (−3.29, 1.47)	−1.18 (−3.45, 1.10)	−1.36 (−4.07, 1.35)	−1.65 (−4.21, 0.90)

*Match preferences*				
Yes cf. no^b^	0.78 (−0.07, 1.62)	0.52 (−0.40, 1.45)	0.13 (−0.82, 1.08)	−0.30 (−1.33, 0.72)

*Treatment expectations*				
High (8–10) cf. low (1–7) general expectation^b^	0.90 (0.05, 1.75)	0.46 (−0.37, 1.30)	1.22 (0.28, 2.17)	0.68 (−0.24, 1.59)
High (6–10) cf. low (1–5) expectation for A&E^b^	0.72 (−0.14, 1.57)	0.54 (−0.28, 1.36)	0.74 (−0.22, 1.70)	0.49 (−0.43, 1.40)
High (6–10) cf. low (1–5) expectation for acupuncture^b^	1.20 (0.33, 2.07)	0.70 (−0.17, 1.57)	1.30 (0.31, 2.28)	0.64 (−0.34, 1.62)

*Matched treatment expectations*^c^				
Yes cf. no^b^	1.14 (0.28, 2.01)	0.66 (−0.20, 1.51)	1.34 (0.36, 2.32)	0.71 (−0.25, 1.67)

a Adjusted for baseline WOMAC pain, age, gender, duration of knee problems, and randomised treatment group.

b Compared to a change of 0 in the comparison group.

c Using cut-offs (at median) to define high/low treatment expectations.

In total, 163 participants were matched for their treatment preference, i.e. they stated a preference for the treatment they were randomised to. The degree of matching was similar for the two acupuncture groups (A&E plus true acupuncture *n* = 70 and A&E plus non-penetrating acupuncture *n* = 78) but was much lower for advice and exercise alone (*n* = 15). Patients who received the treatment for which they had stated a preference did not obtain greater reductions in pain (see Table 3).

**Table 3:** Association of patients baseline treatment preferences and expectations with OMERACT-OARSI responder.

	OMERACT-OARSI at 6 months		OMERACT-OARSI at 12 months	
	Unadjusted odds ratio (95% CI)	Adjusted^a^ odds ratio (95% CI)	Unadjusted odds ratio (95% CI)	Adjusted^a^ odds ratio (95% CI)
*Preference for treatment received*				
Yes cf. no^b^	1.48 (0.86, 2.55)	1.76 (1.00, 3.10)	0.98 (0.57, 1.70)	1.09 (0.61 1.93)

*Preference for A&E*				
Strongly prefer/prefer cf. no preference^b^	1.09 (0.70, 1.72)	1.20 (0.74, 1.94)	1.17 (0.73, 1.86)	1.19 (0.72, 1.96)
Strongly not prefer/not prefer cf. no preference^b^	0.81 (0.32, 2.03)	0.70 (0.27, 1.82)	0.54 (0.20, 1.44)	0.50 (0.18, 1.41)

*Preference for acupuncture*				
Strongly prefer/prefer cf. no preference^b^	1.14 (0.73, 1.78)	1.21 (0.75, 1.96)	0.86 (0.54, 1.36)	0.91 (0.55, 1.51)
Strongly not prefer/not prefer cf. no preference^b^	0.63 (0.18, 2.26)	0.58 (0.15, 2.24)	0.69 (0.20, 2.38)	0.63 (0.17, 2.38)

*Matched preferences*				
Yes cf. no^b^	1.42 (0.92, 2.20)	1.33 (0.79, 2.24)	0.91 (0.58, 1.42)	0.82 (0.48, 1.40)

*Treatment expectations*				
High (8–10) cf. low (1–7) general expectation	1.91 (1.23, 2.97)	1.79 (1.12, 2.87)	1.81 (1.15, 2.86)	1.62 (1.00, 2.63)
High (6–10) cf. low (1–5) expectation for A&E	1.66 (1.06, 2.59)	1.57 (0.99, 2.49)	1.72 (1.09, 2.72)	1.59 (0.99, 2.58)
High (6–10) cf. low (1–5) expectation for acupuncture	1.70 (1.07, 2.69)	1.68 (1.03, 2.75)	1.69 (1.05, 2.71)	1.75 (1.04, 2.93)

*Matched treatment expectations*				
Yes cf. no^c^	1.81 (1.15, 2.87)	1.72 (1.06, 2.79)	1.91 (1.19, 3.06)	1.88 (1.13, 3.13)

a Adjusted for baseline WOMAC pain, age, gender, duration of knee problems, and randomised treatment group.

b Compared to an odds ratio of 1 in the comparison group.

c Using cut-offs (at median) to define high/low treatment expectations.

In total, 202 participants were matched for their treatment expectation, ie they gave a high score (6–10) for the treatment they were randomised to. The degree of matching was similar for those randomised to advice and exercise (*n* = 65), A&E plus true acupuncture (*n* = 70), and A&E plus non-penetrating acupuncture (*n* = 67). There was no relationship between general outcome expectations at baseline and pain reduction at 6 and 12 months.

Patients who received the treatment for which they had expressed high expectations did not have lower pain scores over time. When treatment response was considered using a secondary outcome, the OMERACT-OARSI responder criteria, those patients who received the treatment for which they had high expectations of benefit were more likely to be classified as a treatment responder at 6 months (adjusted odds ratio (OR) 1.72 (1.06, 2.79)) and 12 months (adjusted OR 1.88 (1.13, 3.13)).

### 3.2 Physiotherapists’ expectations and preferences

Physiotherapists expressed a treatment preference for 43% of patients. Overall, the physiotherapists had high expectations of improvement for their patients, reporting a mean general outcome expectation of 6.6 (2.0) out of 10. They had similar expectations of benefit from advice and exercise (6.0 (1.8)) and acupuncture (5.8 (1.9)), with little difference between the three trial arms (Table S2).

### 3.3 Matched patient and physiotherapist treatment expectations and preferences

Table 4 presents the predictive ability of matched treatment expectations and preferences (the match of both patient and therapist) for outcome at 6 and 12 months as measured by the WOMAC pain scale. In total, 51 patients were matched for both their own and their physiotherapist’s treatment preference, ie both the patient and their physiotherapist gave a preference for the treatment they were randomised to. The degree of matching was similar for the two acupuncture groups (A&E plus true acupuncture *n* = 21 and A&E plus non-penetrating acupuncture *n* = 24) but was lower for advice and exercise alone (*n* = 6). There was no relationship between matching the patients’ and physiotherapists’ preference with the treatment received and patients’ clinical outcome.

**Table 4:** Association of baseline treatment expectations and preferences with change in WOMAC pain score and OMERACT-OARSI responder at follow-up: matched for both participant and physiotherapist.

	Change in WOMAC pain at 6 months		Change in WOMAC pain at 12 months	
	Unadjusted mean difference (95% CI)	Adjusted^a^ mean difference (95% CI)	Unadjusted mean difference (95% CI)	Adjusted^a^ mean difference (95% CI)
*Matched patient and therapist treatment preferences*				
Yes cf. no^b^	−0.57 (−1.75, 0.62)	−0.92 (−2.07, 0.23)	−0.27 (−1.62, 1.08)	−0.71 (−2.00, 0.59)
*Matched patient and therapist treatment expectations*^c^				
Yes cf. no^b^	1.21 (0.32, 2.10)	0.82 (−0.05, 1.69)	1.33 (0.33, 2.34)	0.92 (−0.05, 1.89)

	Treatment responder at 6 months		Treatment responder at 12 months	
	Unadjusted odds ratio (95% CI)	Adjusted^a^ odds ratio (95% CI)	Unadjusted odds ratio (95% CI)	Adjusted^a^ odds ratio (95% CI)
*Matched patient and therapist treatment preferences*				
Yes cf. No^d^	0.92 (0.50, 1.70)	0.88 (0.46, 1.68)	0.77 (0.41, 1.45)	0.70 (0.36, 1.38)
*Matched patient and therapist treatment expectations*^c^				
Yes cf. no^d^	1.58 (0.99, 2.52)	1.40 (0.86, 2.28)	1.62 (1.00, 2.62)	1.52 (0.91, 2.53)

a Adjusted for baseline WOMAC pain, age, gender, duration of knee problems, and randomised treatment group.

b Compared to a change of 0 in the not matched group.

c Using cut-offs (at median) to define high/low treatment expectations.

d Compared to an odds ratio of 1 in the comparison group.

120 Patients were matched for both their own and their physiotherapist’s treatment expectation, ie both the patient and their physiotherapist gave a high expectation score for the treatment they were randomised to. The degree of matching was similar for those randomised to advice and exercise (A&E) (*n* = 41), A&E plus true acupuncture (*n* = 40), and A&E plus non-penetrating acupuncture (*n* = 39). There was no clear evidence that when patients received the treatment for which both they, and their therapist, held high expectations, that the change in pain was greater than when there was no match.

## 4 Discussion

### 4.1 Summary and interpretation

In this trial of exercise and acupuncture for patients with knee osteoarthritis, and using our primary outcome (WOMAC pain), we found no relationship between patients’ or practitioners’ treatment preferences and clinical outcome at 6 or 12 months follow-up. We also found no relationship between patients’ general outcome expectations or specific-treatment expectations and their pain levels at 6 and 12 months.

We found some evidence, using a secondary outcome measure, that patients who held high general expectations about the future of their knee problem were more likely to be classified as a treatment responder at 6 and 12 months, than those with low expectations (with odds ratios of 1.79 at 6 months and 1.62 at 12 months). Using the same secondary outcome measure, patients who received the treatment for which they held high expectations were more likely, than those who did not, to be classified as a treatment responder at 6 and 12 months (with odds ratios of 1.72 and 1.88, respectively). Patients who received the treatment for which they had high expectations of benefit were almost twice as likely to be classified as a treatment responder 6 and 12 months later. Asking patients to rate their overall evaluation of change since baseline, one of the criteria for the OMERACT-OARSI response outcome, may be tapping into constructs such as the patients’ satisfaction with treatment or practitioner. This trial also showed, albeit in analyses that are somewhat underpowered, that if patients received the treatment for which both they and their physiotherapist had high expectations, no additional benefit in treatment response was achieved over a match for the patient alone.

Our primary analysis from the trial concluded that acupuncture may have effects through mechanisms other than the specific needling effects. This paper adds to this by showing no clear relationships between patients’ or therapists’ preferences or expectations and pain reduction (WOMAC pain). There was some evidence of a relationship between patients’ expectations and outcome using a secondary outcome measure only.

It is possible that expectations may contribute to the small additional benefits in pain intensity and unpleasantness seen in the acupuncture groups in the trial (Foster et al., 2007). Patients’ treatment expectations may contribute to treatment response when that treatment is received, because they may serve to enhance motivation and compliance with the treatment, they may predict patient satisfaction with the consultation and their healthcare or, as others (Lurie, 2001, Linde et al., 2007) suggest, they may affect patients’ reports of their health status more than their actual health. Research on the mechanisms of expectation effects emphasise the complexity of mind–body interactions, and the role of multiple pathways, endogenous opioids and other non-opioid mechanisms (Finniss and Benedetti, 2005, Goffaux et al., 2007), plus intermediate processes such as improvements in therapeutic alliance between patient and professional (Price et al., 2008) or changes in patients perceptions of their problem and coping strategies (Johnson et al., 2007).

Our results warrant several observations. Firstly, only 20% of our sample stated a treatment preference at baseline, more non-participants reported specific-treatment preferences and, although they had similar expectations of advice and exercise, they had lower expectations of acupuncture. Although the extent to which the responses from the 94 patients, who did not participate in the trial but who responded to our questions about preferences, are generalisable to the larger group of those who declined to participate is unknown, it does suggest that patients’ treatment preferences and expectations can affect the recruitment to randomised trials. Our results may be specific to the clinical problem of knee osteoarthritis or the nature of the interventions investigated. For example in comparison to exercise, acupuncture is invasive, seen as ‘unusual’ in western healthcare and thus has a flavour of exoticism, which may serve to increase the potential for non-specific effects. Other factors, such as the time spent with practitioners, the characteristics of the practitioner and the practice setting were constant across all interventions and thus cannot explain the effects of expectations seen.

Of note were the high baseline expectations of benefit from all treatments, potentially explained by the fact that all patients had already been referred from their general practitioner to physiotherapy. It is possible that they may have already been influenced by a respected medical opinion of the potential value of physiotherapy treatments.

Our observation that trial physiotherapists had generally similar expectations for advice and exercise or acupuncture, may have several explanations. For example, it is possible this is a true picture of physiotherapists’ views of these treatments as our results were based on large numbers across a wide geographical area in the UK. However, it could be that only therapists with generally equal expectations of both types of treatment participated. The lack of associations between therapists’ preferences and expectations and clinical outcomes may have been due to therapists changing their usual interactions to deliberately avoid promoting one treatment over another in the context of a trial. If so, the role of therapist preference and expectations effects on outcome might be better investigated within observational studies.

### 4.2 Comparison with other studies

Our results support those of King et al. (2005) who showed that participants’ preferences may affect trial recruitment but have no clear effect on outcomes. They are also in line with those from an individual patient data meta-analysis of 11 trials (Preference Collaborative Review Group, 2008) that showed no demographic or clinical differences between trial participants with, and without, a preference. Yet our results differ with the conclusions of that meta-analysis as we have shown no clear relationship between patients’ preferences and outcomes. Others have shown mixed results ranging from no effects of patient preferences (Moffett et al., 1999), no effects of patient or practitioner preferences (Stewart et al., 2008), positive associations between patient preferences and outcomes (Thomas et al., 2004, Johnson et al., 2007) and counter-intuitive effects, where patients who expressed expectations of benefit from acupuncture had poorer outcomes than those who were not sure (Thomas et al., 2006).

We did not find a clear relationship between patients’ expectations and pain reduction using our primary outcome measure, but the results from our secondary outcome support previous studies (Kalauokalani et al., 2001, Mondloch et al., 2001, Charron et al., 2006, Linde et al., 2007) that conclude relationships between patients’ general recovery and treatment expectations and their outcomes. Linde et al. (2007) showed that patients with high expectations were twice as likely to respond well to treatment as those with low expectations, a finding of similar size to our own.

Comparing our results of therapists’ preferences and expectations is difficult since the available systematic review (King et al., 2005) included only three trials examining physicians’ preferences and, in all, the outcome was in favour of the preference arms. Only one previous study investigated physiotherapists’ preferences (Stewart et al., 2008) and found they did not moderate the effect of treatment.

### 4.3 Strengths and weaknesses

The strengths of this research include the preferences and expectations data of patients who were approached to take part in the trial, but who declined to participate. We measured both patients’ and therapists’ expectations and preferences and explored the potential importance of a match between these. We tried to improve on the information previously available in trials, by capturing information on the strength of preferences and expectations. We followed patients up at 6 and 12 months and showed reasonably consistent results at both time-points. The limitations include the relatively small sample size for these secondary analyses, since the main aim of the trial was to determine the effectiveness of the treatments. It is currently not known how best to elicit or measure patient or clinician preferences and expectations. Like this trial, most studies have previously used either simple numerical rating scales or non-validated measurement tools, and some have adapted measures from questionnaires originally developed for patients, for use with clinicians. A recent study has highlighted that the development and testing of such tools is in its infancy (Bishop et al., 2007).

### 4.4 Implications

Patients’ treatment preferences and expectations were not associated with the change in their knee pain, the primary outcome, at 6 and 12 months follow-up. Their general outcome expectations and specific-treatment expectations were associated with a secondary outcome, of treatment response, but they are clearly only one of a multiplicity of factors influencing outcome. As previously advocated (Kravitz, 2001), learning to elicit, evaluate and include patients’ expectations about their pain and about treatments available may be potentially useful to optimise treatment response. This information might be particularly important where there are multiple treatment options with little differences in effectiveness as is the case for many common musculoskeletal problems.

## 5 Conclusions

We found no evidence of a relationship between patients’ treatment preferences or expectations and pain reduction. We found weak evidence, with a secondary outcome, that knee pain patients’ expectations, both general and treatment-specific, are related to clinical outcome from exercise and acupuncture. Considering the practitioners’ preferences and expectations for individual patients within the trial did not add further explanation of outcomes.

## Appendix A Supplementary material

Table S1 Patients baseline treatment choice, preference and expectation data.

Table S2 Physiotherapists’ treatment expectations and preferences.

Appendices 1 and 2

## References

[bib1] Bausell R.P., Lao L., Bergman S., Berman B.M. (2005). Is acupuncture analgesia an expectancy effect?. Eval Health Prof.

[bib2] Bellamy N. WOMAC Osteoarthritis Index. A User’s Guide, Ontario; 1996.

[bib3] Bishop A., Thomas E., Foster N.E. (2007). Health care professionals’ attitudes and beliefs about low back pain: a systematic search and critical review of available measurement tools. Pain.

[bib4] Bishop A., Foster N.E., Thomas E., Hay E.M. (2008). How does the self-reported clinical management of patients with low back pain relate to the attitudes and beliefs of health care practitioners? A survey of UK general practitioners and physiotherapists. Pain.

[bib5] Charron J., Rainville P., Marchand S. (2006). Direct comparison of placebo effects on clinical and experimental pain. Clin J Pain.

[bib6] Coudeyre E., Rannou F., Tubach F., Baron G., Coriat F., Brin S. (2006). General practitioners’ fear-avoidance beliefs influence their management of patients with low back pain. Pain.

[bib7] Crow R., Gage H., Hampson S., Hart J., Kiimber A., Thomas H. (1999). The role of expectancies in the placebo effect and their use in the delivery of health care: a systematic review. Health Technol Assess.

[bib8] Di Blasi Z., Harkness E., Ernst E., Georgiou A., Kleijnen J. (2001). Influence of context effects on health outcomes: a systematic review. Lancet.

[bib9] Ernst E., Peters D. (2001). Understanding the placebo effect in complementary medicine: theory, practice and research.

[bib10] Ersek M., Turner J.A., McCurry S.M., Gibbobs L., Miller-Kraybill B. (2003). Efficacy of a self-management group intervention for elderly persons with chronic pain. Clin J Pain.

[bib11] Feinstein A.R. (2002). Post-therapeutic response and therapeutic style: re-formulating the placebo effect. J Clin Epidemiol.

[bib12] Finniss D.G., Benedetti F. (2005). Mechanisms of the placebo response and their impact on clinical trials and clinical practice. Pain.

[bib13] Foster N.E. (2007). Beliefs and preferences: do they help determine the outcome of musculoskeletal problems?. Phys Ther Rev.

[bib14] Foster N.E., Thomas E., Barlas P., Hill J.C., Young J., Mason E. (2007). Acupuncture as an adjunct to exercise based physiotherapy for osteoarthritis of the knee: randomised controlled trial. BMJ.

[bib15] Galer B.S., Schwartz L., Turner J.A. (1997). Do patient and physician expectations predict response to pain-relieving procedures?. Clin J Pain.

[bib16] Goffaux P., Redmond W.J., Rainville P., Marchand S. (2007). Descending analgesia – when the spine echoes what the brain expects. Pain.

[bib17] Hay E., Barlas P., Foster N., Hill J., Thomas E., Young J. (2004). Is acupuncture a useful adjunct to physiotherapy for older adults with knee pain?: the “acupuncture, physiotherapy and exercise” (APEX) study [ISRCTN88597683]. BMC Musculoskelet Disord.

[bib18] Heymans M.W., de Vet H.C.W., Knol D.L., Bongers P., Koes B.W., van Mechelen W. (2006). Workers’ beliefs and expectations affect return to work over 12 months. J Occup Rehabil.

[bib19] Johnson R.E., Jones G.T., Wiles N.J., Chaddock C., Potter R.G., Roberts C. (2007). Active exercise, education and cognitive behavioral therapy for persistent disabling low back pain: a randomised controlled trial. Spine.

[bib20] Kalauokalani D., Cherkin D.C., Sherman K.J., Koepsell T.D., Deyo R.A. (2001). Lessons from a trial of acupuncture and massage for low back pain: patient expectations and treatment effects. Spine.

[bib21] Kaptchuk T.J., Kelley J.M., Conboy L.A., Davis R.B., Kerr C.E., Jacobson E.E. (2008). Components of the placebo effect: randomised controlled trial in patients with irritable bowel syndrome. BMJ.

[bib22] King M., Nazareth I., Lampe F., Bower P., Chandler M., Morou M. (2005). Conceptual framework and systematic review of the effects of participants’ and professionals’ preferences in randomised controlled trials. Health Technol Assess.

[bib23] Klaber-Moffett J.A., Jackson D.A., Richmond S., Hahn S., Coulton S., Farrin A. (2005). Randomised trial of brief physiotherapy intervention compared with usual physiotherapy for neck pain patients: outcomes and patients preference. BMJ.

[bib24] Kravitz R.L. (2001). Measuring patients’ expectations and requests. Ann Intern Med.

[bib25] Linde K., Witt C.M., Streng A., Weidenhammer W., Wagenpfeil S., Brinkhasu B. (2007). The impact of patient expectations on outcomes in four randomized controlled trials of acupuncture in patients with chronic pain. Pain.

[bib26] Lurie J.D. (2001). Point of view. Spine.

[bib27] Manheimer E., Linde K., Lao L., Bouter L.M., Berman B.M. (2007). Meta-analysis: acupuncture for osteoarthritis of the knee. Ann Intern Med.

[bib28] Moffett J.K., Torgerson D., Bell-Syer S., Jackson D., Llewlyn-Phillips H., Farrin A. (1999). Randomised controlled trial of exercise for low back pain: clinical outcomes, costs and preferences. BMJ.

[bib29] Mondloch M.V., Cole D.C., Frank J.W. (2001). Does how you do depend on how you think you’ll do? A systematic review of the evidence for a relation between patients’ recovery expectations and health outcomes. CMAJ.

[bib30] Montgomery A.A., Fahey T. (2001). How do patients’ treatment preferences compare with those of clinicians?. Qual Health Care.

[bib31] Office for National Statistics (2000).

[bib32] Office for National Statistics (2002).

[bib33] Parsons S., Harding G., Breen A., Foster N., Pincus T., Vogel S. (2007). The influence of patients’ and primary care practitioners’ beliefs and expectations about chronic musculoskeletal pain on the process of care: a systematic review of qualitative studies. Clin J Pain.

[bib34] Pham T., Van der Heijde D., Lassere M., Altman R.D., Anderson J.J., Bellamy N. (2003). Outcome variables for osteoarthritis clinical trials: the OMERACT-OARSI set of responders criteria. J Rheumatol.

[bib35] Pham T., Van der Heijde D., Altman R.D., Anderson J.J., Bellamy N., Hochberg M. (2004). OMERACT-OARSI Initiative: Osteoarthritis Research Society International set of responder criteria for osteoarthritis clinical trials revisited. Osteoarthr Cartilage.

[bib36] Pincus T., Vogel S., Santos R., Breen A., Foster N., Underwood M. (2006). The attitudes to back pain scale in musculoskeletal practitioners (ABS-mp): the development and testing of a new questionnaire. Clin J Pain.

[bib37] Pincus T., Foster N.E., Vogel S., Santos R., Breen A., Underwood M. (2007). Attitudes to back pain amongst musculoskeletal practitioners: a comparison of professional groups and practice settings using the ABS-mp. Manual Ther.

[bib38] Poiraudeau S., Rannou F., Henanff L.E., Coudeyre E., Rozenberg S., Huas D. (2006). Outcome of subacute low back pain: influence of patients’ and rheumatologists’ characteristics. Rheumatol.

[bib39] Preference Collaborative Review Group (2008). Patients’ preferences within randomised trials: systematic review and patient level meta-analysis. BMJ.

[bib40] Price M., Anderson P., Henrich C.C. (2008). Greater expectations: using hierarchical linear modelling to examine expectancy for treatment outcome as a predictor of treatment response. Behav Ther.

[bib41] Stewart M.J., Maher C.G., Refshauge K.M., Herbert R.D., Nicholas M.K. (2008). Patient and clinician treatment preferences do not moderate the effect of exercise treatment in chronic whiplash-associated disorders. Eur J Pain.

[bib42] Streitberger K., Kleinhenz J. (1998). Introducing a placebo needle into acupuncture research. Lancet.

[bib43] Thomas E., Croft P.R., Paterson S.M., Dziedzic K., Hay E.M. (2004). What influences participants’ treatment preference and can it influence outcome? Results from a primary care-based randomised trial for shoulder pain. Br J Gen Pract.

[bib44] Thomas K.J., MacPherson H., Thorpe L., Brazier J., Fitter M., Campbell M.J. (2006). Randomised controlled trial of a short course of traditional acupuncture compared with usual care for persistent non-specific low back pain. BMJ.

[bib45] Turner J.A., Jensen M.P., Warms C.A., Cardenas D.D. (2002). Blinding effectiveness and association of pre-treatment expectations with pain improvement in a double-blind randomized controlled trial. Pain.

[bib46] Van der Windt D.A., Koes B.W., van Aarst M., Heemskerk M.A., Bouter L.M. (2000). Practical aspects of conducting a randomised pragmatic trial in primary care: patient recruitment and outcome assessment. Br J Gen Pract.

