# Supplementary material for: The relationship between patient and practitioner expectations and preferences and clinical outcomes in a trial of exercise and acupuncture for knee osteoarthritis
Source: Eur J Pain. 2010 Apr;14(4):402–9. doi: 10.1016/j.ejpain.2009.06.010 (PMC2856919; doi:10.1016/j.ejpain.2009.06.010)
Supplement: Table S1 — Patients baseline treatment choice, preference and expectation data. [file mmc1.doc]

**Table S1 – Patients baseline treatment choice, preference and expectation data**

|  | Advice & exercise (A&E)  (n=116) | A&E +  true acupuncture  (n=117) | A&E +  non-penetrating acupuncture  (n=119) |
| --- | --- | --- | --- |
| Treatment choice |  |  |  |
| *‘If you had a free choice which treatment would you choose’* Advice and Exercise (A&E)  Acupuncture  A&E and acupuncture  Injection  Diet  Surgery  No preference | 15 (13%)  12 (11%)  43 (37%)  1 ( 1%)  0 ( 0%)  0 ( 0%)  44 (38%) | 10 ( 9%)  18 (15%)  52 (44%)  0 ( 0%)  1 ( 1%)  1 ( 1%)  35 (30%) | 10 ( 9%)  17 (14%)  61 (51%)  0 ( 0%)  0 ( 0%)  1 ( 1%)  29 (25%) |
| Preferences |  |  |  |
| Strength of preference for A&E Strongly prefer  Prefer  No preference  Not prefer  Strongly not prefer | 24 (21%)  21 (19%)  61 (54%)  6 ( 5%)  1 ( 1%) | 18 (16%)  42 (36%)  48 (41%)  6 ( 5%)  2 ( 2%) | 29 (24%)  23 (19%)  58 (49%)  8 ( 7%)  1 ( 1%) |
| Strength of preference for Acupuncture Strongly prefer  Prefer  No preference  Not prefer  Strongly not prefer | 19 (17%)  30 (26%)  62 (55%)  2 ( 2%)  0 ( 0%) | 14 (12%)  43 (38%)  51 (45%)  5 ( 5%)  0 ( 0%) | 23 (19%)  41 (34%)  50 (42%)  3 ( 3%)  2 ( 2%) |
| Expectations |  |  |  |
| General outcome expectation*  *“How hopeful are you that your knee problem will get better”* | 7.5 (2.4) | 7.0 (2.3) | 7.2 (2.5) |
| Expect A&E to help knee pain  Of great help  Of some help  Of little help  Of no help | 42 (37%)  63 (54%)  9 ( 8%)  1 ( 1%) | 36 (31%)  75 (64%)  4 ( 3%)  2 ( 2%) | 36 (30%)  67 (56%)  10 ( 9%)  6 ( 5%) |
| Strength of expectation with A&E* | 5.8 (2.5) | 6.1 (2.1) | 5.6 (2.4) |
| Expect Acupuncture to help knee pain  Of great help  Of some help  Of little help  Of no help | 41 (37%)  65 (59%)  4 ( 3%)  1 ( 1%) | 36 (31%)  71 (61%)  8 ( 7%)  1 ( 1%) | 53 (46%)  58 (50%)  5 ( 4%)  0 ( 0%) |
| Strength of expectation with Acupuncture* | 6.1 (2.3) | 6.4 (2.2) | 6.4 (2.2) |

* - Data are mean (standard deviation) from a 0-10 numerical rating scale
