# Supplementary material for: The relationship between patient and practitioner expectations and preferences and clinical outcomes in a trial of exercise and acupuncture for knee osteoarthritis
Source: Eur J Pain. 2010 Apr;14(4):402–9. doi: 10.1016/j.ejpain.2009.06.010 (PMC2856919; doi:10.1016/j.ejpain.2009.06.010)
Supplement: Table S2 — Physiotherapists’ treatment expectations and preferences. [file mmc2.doc]

**Table S2 – Physiotherapists’ treatment expectations and preferences**

|  | Advice & exercise (A&E)  (n=116) | A&E +  true acupuncture  (n=117) | A&E +  non-penetrating acupuncture  (n=119) |
| --- | --- | --- | --- |
| Preferences |  |  |  |
| Have a treatment preference No  Yes | 65 (57%)  50 (43%) | 72 (62%)  45 (38%) | 62 (52%)  57 (48%) |
| *‘If you had a free choice which treatment would you choose’* Advice and Exercise  Acupuncture  Advice, exercise and acupuncture  Other physiotherapy treatment  Surgery  No preference | 56 (49%)  34 (30%)  4 ( 3%)  1 ( 1%)  1 ( 1%)  18 (16%) | 59 (49%)  25 (22%)  3 ( 3%)  3 ( 3%)  0 ( 0%)  27 (23%) | 55 (46%)  36 (30%)  5 ( 4%)  3 ( 3%)  1 ( 1%)  19 (16%) |
| Expectations |  |  |  |
| General outcome expectation*  *“How hopeful are you that the patient’s knee problem will get better”* | 6.6 (1.9) | 6.8 (2.1) | 6.5 (2.1) |
| Expect A&E to help knee pain  Of great help  Of some help  Of little help  Of no help | 26 (23%)  72 (62%)  14 (12%)  3 ( 3%) | 39 (33%)  64 (55%)  14 (12%)  0 ( 0%) | 29 (24%)  80 (67%)  9 ( 8%)  1 ( 1%) |
| Strength of expectation with A&E* | 5.9 (1.8) | 6.1 (1.9) | 6.1 (1.8) |
| Expect Acupuncture to help knee pain  Of great help  Of some help  Of little help  Of no help | 43 (37%)  62 (54%)  9 ( 8%)  1 ( 1%) | 42 (36%)  63 (54%)  11 ( 9%)  1 ( 1%) | 41 (34%)  71 (60%)  7 ( 6%)  0 ( 0%) |
| Strength of expectation with Acupuncture* | 5.9 (1.8) | 5.7 (2.0) | 5.8 (2.0) |

* - Data are mean (standard deviation) from a 0-10 numerical rating scale
