# Supplementary material for: The relationship between patient and practitioner expectations and preferences and clinical outcomes in a trial of exercise and acupuncture for knee osteoarthritis
Source: Eur J Pain. 2010 Apr;14(4):402–9. doi: 10.1016/j.ejpain.2009.06.010 (PMC2856919; doi:10.1016/j.ejpain.2009.06.010)
Supplement: Appendices 1 and 2 [file mmc3.doc]

Appendix 1 Questions to patients

**1.** Please put a cross through the number that best describes how hopeful you are that your knee problem will get better.

Not hopeful at all

Extremely hopeful

| 0 | 1 | 2 | 3 | 4 | 5 | 6 | 7 | 8 | 9 | 10 |
| --- | --- | --- | --- | --- | --- | --- | --- | --- | --- | --- |

Before the study begins, we want to know about your preferences for the different treatments you may receive as part of this study.

1. Do you have a preference for the type of treatment you receive?

**Yes No**

1. If you had a free choice, which treatment would you choose for your knee problem? (Please put a cross in one box only)

Advice and exercise………….……………

Acupuncture………………………………

Both together……………………………..

No preference……………………………..

Other (please give details)________________________________

1. Please indicate how strongly you would prefer the treatment which includes: (Please put a cross in one box only)

a) Advice and exercises?

**Strongly not prefer**

**Strongly prefer Prefer No preference Not prefer**

b) Acupuncture?

**Strongly not prefer**

**Strongly prefer Prefer No preference Not prefer**

The following questions are about your expectations about different treatments.

##### On a scale where 0 is no change at all and 10 is completely better, please put a cross through the number which best describes how much you would expect your knee problem to improve with each of the following treatments:

a) Advice and exercises

No change at all

Completely better

| 0 | 1 | 2 | 3 | 4 | 5 | 6 | 7 | 8 | 9 | 10 |
| --- | --- | --- | --- | --- | --- | --- | --- | --- | --- | --- |

b) Acupuncture

No change at all

Completely better

| 0 | 1 | 2 | 3 | 4 | 5 | 6 | 7 | 8 | 9 | 10 |
| --- | --- | --- | --- | --- | --- | --- | --- | --- | --- | --- |

To help us find out about your specific expectations about treatment for your knee, the next 2 questions ask about knee pain, movement and function. (Please put a cross in one box on each line)

1. Please indicate how much you expect advice and exercises to help:

Of great help

Of some help

Of little help

Of no help

**Your knee pain**

**Your knee movement**

**Your knee function**

1. Please indicate how much you expect acupuncture to help:

Of little help

Of no help

Of great help

Of some help

**Your knee pain**

**Your knee movement**

**Your knee function**

Appendix 2 Questions to physiotherapists during randomisation telephone call

We want to know about your treatment preferences for the individual patients you are seeing as part of this study. These questions ask about your preferences and expectations about specific treatments. We would like you to answer these questions with your specific patient in mind.

1. Do you have a preference for which treatment this patient receives?

Yes No

2. If you had a free choice, which treatment would you choose for this knee patient? *(Please select one option only*)

Advice and exercise………….………………..

Acupuncture…………………………………..

No preference………………………………….

##### 3. On a scale where 0 is no change at all and 10 is completely better, please select the number which best describes how much you expect this patient’s knee problem to improve with advice and exercise (where 0 is ‘no change at all’ and 10 is ‘completely better’)

###### No change at all

###### Completely better

| 0 | 1 | 2 | 3 | 4 | 5 | 6 | 7 | 8 | 9 | 10 |
| --- | --- | --- | --- | --- | --- | --- | --- | --- | --- | --- |

4. How much do you expect advice and exercises to help this patient’s

Of great help

###### Of some help

###### Of little help

###### Of no help

knee pain

knee movement

knee function

##### 5. On a scale where 0 is no change at all and 10 is completely better, please select the number which best describes how much you expect this patient’s knee problem to improve with acupuncture treatment (where 0 is ‘no change at all’ and 10 is ‘completely better’)

###### No change at all

###### Completely better

| 0 | 1 | 2 | 3 | 4 | 5 | 6 | 7 | 8 | 9 | 10 |
| --- | --- | --- | --- | --- | --- | --- | --- | --- | --- | --- |

6. How much do you expect acupuncture to help this patient’s

Of great help

###### Of some help

###### Of little help

###### Of no help

knee pain

knee movement

knee function

7. On a scale where 0 is not hopeful at all and 10 is extremely hopeful, please select the number which best describes how hopeful you are that this patient’s knee problem will get better.

Not hopeful at all

Extremely hopeful

| 0 | 1 | 2 | 3 | 4 | 5 | 6 | 7 | 8 | 9 | 10 |
| --- | --- | --- | --- | --- | --- | --- | --- | --- | --- | --- |
